# Safety and Efficacy of Pulsed Field Ablation for Atrial Fibrillation in Older Patients: An Observational Study at a Large Tertiary Centre in Australia

**DOI:** 10.1002/joa3.70336

**Published:** 2026-04-06

**Authors:** Francis J. Ha, Duron Prinsloo, Hui Chen Han, Nitesh Nerlekar, Adam J. Brown, Emily Kotschet

**Affiliations:** ^1^ Victorian Heart Institute, Victorian Heart Hospital Monash University Clayton Victoria Australia

**Keywords:** atrial fibrillation, catheter ablation, elderly

## Abstract

**Background:**

Older patients have been under‐represented in clinical trials of atrial fibrillation (AF) ablation. The safety of pulsed field ablation (PFA) in this cohort is not known.

**Methods:**

We conducted an observational study of consecutive patients undergoing PFA with a pentaspline catheter (Farapulse, Boston Scientific) for AF at a tertiary centre comparing procedural and clinical outcomes in patients aged ≥ 75 years (older cohort) versus < 75 years (younger cohort). Baseline demographics, procedural characteristics, complications, and clinical outcomes were collected. Arrhythmia recurrence was defined as AF, atrial flutter, or atrial tachycardia > 30 s at any follow‐up.

**Results:**

564 consecutive patients underwent de novo PFA for AF between 2022 and 2025; 65 patients were aged ≥ 75 years (11.5%) (median age 77 years; younger cohort median age 62 years). The older cohort had a higher incidence of hypertension, vascular disease, lower eGFR, and lower body mass index (*p* < 0.01 for all). There was no difference in lesion set or procedural time. There was no difference in overall major procedural complications (1.5%–1.6%; *p* = 0.97); however, older patients had more major vascular complications (3.1% vs. 0.2%; *p* = 0.003), pulmonary oedema (3.1% vs. 0.4%; *p* = 0.02), and transient renal impairment when assessed (*p* = 0.007). At median follow‐up 6.3–7.0 months, there was no difference in freedom from arrhythmia recurrence between the older and younger cohorts (69.8% and 73.2%; *p* = 0.66).

**Conclusion:**

Pulsed field ablation for AF can be performed in older patients with acceptable procedural safety and clinical outcomes. Clinical trials are needed to further determine the safety and efficacy of PFA in older patients.

## Graphical abstract

In this retrospective observational study, pulsed field ablation using a specific pentaspline catheter showed comparable procedural and clinical outcomes between older (aged ≥ 75 years) and younger patients (aged < 75 years). However, judicious patient selection is still warranted and randomized trial data are needed to confirm these findings.

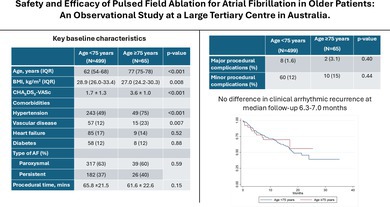

## 1 Introduction

Atrial Fibrillation (AF) is the most prevalent arrhythmia worldwide, with a well‐established association between advancing age and disease burden [1, 2]. Catheter ablation holds a class IA indication for achieving improved rhythm control in symptomatic, drug‐intolerant paroxysmal or persistent AF by reducing recurrence and overall arrhythmic burden while improving quality of life [3, 4, 5]. Thermal ablation techniques such as radiofrequency (RFA) and cryoballoon ablation have been employed however can be limited by their non‐selective nature that poses risk of collateral injury including pulmonary vein stenosis, atrio‐esophageal fistula, and phrenic nerve injury [6, 7, 8].

**FIGURE 1:**
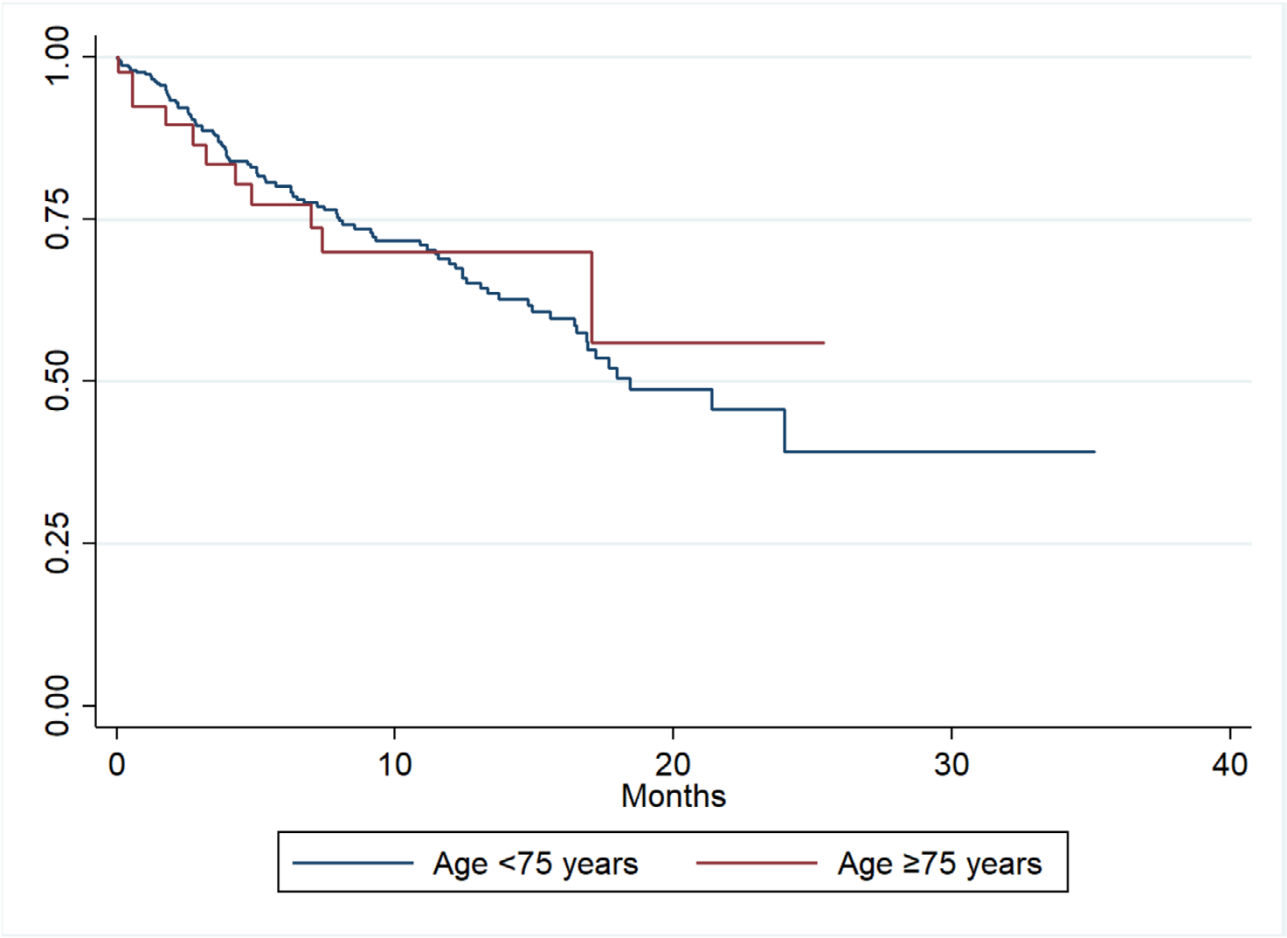
Freedom from arrhythmia recurrence between older and younger cohort.

Pulsed field ablation (PFA) is an emerging non‐thermal ablation that induces irreversible electroporation to more selectively ablate cardiac tissue, offering high tissue specificity while also demonstrating non‐inferior efficacy compared to thermal ablative techniques [9, 10, 11]. Additionally, PFA offers notable procedural efficiency, with comparatively shorter left atrial dwell times and overall procedure duration [6]. This combination of myocardial selectivity and procedural efficiency aids the favorable safety profile observed in early clinical data, with lower rates of non‐myocardial complications [11, 12, 13].

Landmark trials have established the efficacy and safety of catheter ablation in atrial fibrillation; however, there are limited data regarding safety in older patient populations (≥ 75 years), with most studies enrolling cohorts with median ages below 70 or explicitly excluding older individuals [5, 11, 14]. Consequently, real‐world data on the safety of PFA in this demographic remain limited.

Our study aimed to evaluate the safety and efficacy of PFA in patients aged ≥ 75 years undergoing PFA for AF by comparing procedural and clinical outcomes with patients aged < 75 years.

## 2 Methods

We performed a retrospective analysis of consecutive patients who underwent PFA with a pentaspline catheter for AF at an Australian tertiary centre from November 2022 to June 2025. Consecutive patients undergoing de novo PFA were included. Patients were analyzed in two groups based on age < 75 years (younger cohort) or ≥ 75 years (older cohort). Approval for this study was granted by the Monash Health Human Research Ethics Committee.

### 2.1 Baseline Demographics

Baseline demographic data including age, sex, body mass index (BMI), type of AF (paroxysmal or persistent) and medical comorbidities were obtained from electronic medical records. The CHA_2_DS_2_‐VASc score was calculated for each patient and renal function assessed by estimated glomerular filtration rate (eGFR). Left ventricular ejection fraction (LVEF) and indexed left atrial volume (LAVI) were collected where available. Medication data included the type of antiarrhythmic drug and anticoagulant therapy patients were receiving prior to the ablation.

### 2.2 Pulsed Field Ablation Procedure

All patients underwent PFA with the Farapulse system (Boston Scientific, US) under general anesthesia. Cardiac computed tomography was not routinely performed pre‐procedurally. Pulmonary venous anatomy and left atrial appendage assessment was performed using transesophageal echocardiography (TOE) during the procedure. Periprocedural anticoagulation strategy was according to physician preference. Femoral venous access was routinely obtained under both anatomical landmark and ultrasound guidance using a 6Fr and 7Fr short sheath. Transseptal puncture was performed under transesophageal echocardiography and hemodynamic monitoring guidance. Peri‐procedural intravenous heparin was administered to achieve a target activated clotting time (ACT) of 350 s or above. Atropine was administered to minimize the risk of vagal response. The 7Fr femoral sheath was upsized to a 16.8Fr Faradrive sheath. Either a 31 mm or 35 mm pentaspline catheter was selected based on left atrial size. PFA was typically performed on the pulmonary veins in all cases, and ablation of the posterior wall and other non‐pulmonary vein triggers varied according to physician preference. From February 2025 onwards, electroanatomic mapping was concurrently used through an electroanatomic map software integrated into the Farapulse system (Faraview, Boston Scientific, US).

Upon completion of PFA, catheters were removed and femoral closure performed with either figure‐of‐eight suture or rarely a vascular closure device such as Perclose ProStyle devices (Abbott Vascular, USA) according to physician preference. All patients were monitored in the immediate recovery area. Patients underwent same‐day discharge as per standard protocol or were admitted to hospital if necessary or requested by the proceduralist.

Procedural characteristics collected included lesion sets (e.g., pulmonary vein isolation, posterior wall isolation, superior vena cava isolation), total number of lesions, total procedure time, left atrial catheter dwell time, PFA application time (from commencement of PFA application to last application), fluoroscopy time, radiation dose by air kerma (mGy) and dose‐area product (μGym^2^).

### 2.3 Clinical Outcomes

Procedural complications were categorized as major or minor. Major procedural complications included death, atrio‐esophageal fistula, symptomatic pulmonary vein stenosis, cardiac tamponade, stroke, coronary artery spasm, persistent phrenic nerve injury, major vascular complication requiring surgical intervention, and dialysis requirement. Minor procedural complications included systemic air embolism, transient ischaemic attack, transient phrenic nerve injury, minor vascular complication requiring further non‐surgical intervention (e.g., FemoStop application or further manual compression), pericarditis, pulmonary oedema, haemolysis and transient renal impairment (defined as a double in serum creatinine elevation post‐procedure compared with baseline). If patients were admitted to hospital post‐procedure, data were collected regarding day one post‐procedure renal function where available. A routine follow‐up phone call from nursing staff was performed at 7 days post‐procedure, and patients were reviewed in the tertiary centre arrhythmia clinic or with their private cardiologist. Monitoring for arrhythmias at follow‐up was performed based on symptom and clinical indication with standard 12‐lead electrocardiogram (ECG). Arrhythmia recurrence was defined as any AF, atrial flutter or atrial tachycardia lasting more than 30 s at any follow‐up. Detection of arrhythmia occurrence was by 12‐lead ECG if persistent, Holter monitoring or intracardiac device check at follow‐up, or wearable device. Data regarding subsequent need for cardioversion, rehospitalization or need for redo ablation procedure were also collected.

### 2.4 Statistical Analysis

Categorical variables are presented as frequencies and percentages, while continuous variables are reported as median with interquartile range (IQR) or as mean with standard deviation (SD), depending on distribution. Patients were stratified a priori by age (< 75 vs. ≥ 75 years) for comparative analysis. Between‐group comparisons were performed using Pearson's chi‐square test for categorical variables, and student's *t*‐test or analysis of variance (ANOVA) for continuous variables. Kaplan–Meier survival analysis was used to display freedom from arrhythmia recurrence. *p*‐value < 0.05 was considered statistically significant. All analyses were performed using Stata MP 14.0 (StataCorp LP, College Station, TX, USA).

## 3 Results

### 3.1 Baseline Demographics

564 consecutive patients underwent de novo PFA for AF during the study period, of which 65 patients were aged ≥ 75 years (11.5%; Table 1). The median age in the older cohort was 77 years (IQR 75–78) compared with 62 years (IQR 54–68) in the younger cohort (*p* < 0.001). Body mass index was higher in the younger cohort (28.9 vs. 27.0 kg/m^2^, respectively; *p* = 0.008). The CHA_2_DS_2_‐VASc score was higher in the older cohort (mean 3.6 ± 1.0 vs. 1.7 ± 1.3, respectively; *p* < 0.001) with a greater proportion of hypertension (75% vs. 49%; *p* < 0.001), vascular disease (23% vs. 12%; *p* = 0.007) and lower eGFR (66 ± 16 vs. 78 ± 14 mL/min/1.73 m^2^; *p* < 0.001). There was no difference in persistent AF (37%–40%; *p* = 0.59) with similar left ventricular systolic function (LVEF 57%–60%; *p* = 0.44) and LAVI (44.1–45.4 mL/m^2^; *p* = 0.59).

**TABLE 1:** Baseline demographics.

	Age < 75 years	Age ≥ 75 years	
	*N* = 499	*N* = 65	*p*
Age, years (IQR)	62 (54–68)	77 (75–78)	**< 0.001**
Female (%)	172 (35)	24 (37)	0.70
BMI, kg/m^2^ (IQR)	28.9 (26.0–33.4)	27.0 (24.2–30.3)	**0.008**
CHA_2_DS_2_‐VASc	1.7 ± 1.3	3.6 ± 1.0	**< 0.001**
Comorbidities (%)			
Hypertension	243 (49)	49 (75)	**< 0.001**
Vascular disease	57 (12)	15 (23)	**0.007**
Heart failure	85 (17)	9 (14)	0.52
Diabetes mellitus	58 (12)	8 (12)	0.88
Stroke/TIA	25 (5)	2 (3)	0.48
Obstructive sleep apnoea	83 (17)	6 (9)	0.13
eGFR, mL/min/1.73 m^2^	78 ± 14	66 ± 16	**< 0.001**
Type of AF (%)			
Paroxysmal	317 (63)	39 (60)	0.59
Persistent	182 (37)	26 (40)	
Prior AF ablation (%)	63 (13)	9 (14)	0.12
LVEF %, median (IQR)	60 (50–60)	57 (50–60)	0.44
LAVI, ml/m^2^	45.4 ± 13.1	44.1 ± 10.0	0.59
Antiarrhythmic drug			
None	131 (28)	18 (30)	0.76
Flecainide	121 (26)	12 (20)	
Sotalol	143 (30)	18 (30)	
Amiodarone	78 (16)	12 (20)	
Not recorded	26 (5)	5 (7)	
Anticoagulation prior			
None	32 (6)	1 (2)	0.41
Apixaban	355 (72)	47 (73)	
Rivaroxaban	91 (18)	15 (23)	
Dabigatran	11 (2)	1 (2)	
Warfarin	6 (1)	0	
Not recorded	4 (1)	1 (2)	

*Note:* Bold values indicate statistically significant values.

Abbreviations: AF, atrial fibrillation; BMI, Body mass index; IQR, interquartile range; LAVI, left atrial volume indexed; LVEF, left ventricular ejection fraction; TIA, transient ischaemic attack.

### 3.2 Procedural Characteristics

Electroanatomic mapping (Faraview on Rhythmia System, Boston Scientific) was used in 13% of patients across both groups (*p* = 0.94). There was no difference between groups regarding the lesion set performed. Pulmonary vein isolation was routinely performed except for one patient whose veins were isolated from a previous ablation procedure. Posterior wall isolation was performed in 40%–43% of patients (*p* = 0.69) with total applications ranging from median 56–58 applications across both groups (*p* = 0.90). There was no difference in procedural time (61.6–65.8 min; *p* = 0.15), left atrial catheter dwell time (40.9–42.6 min; *p* = 0.44), fluoroscopy time (18.0–19.2 min; *p* = 0.34) or radiation dose. Procedural characteristics are shown in Table 2.

**TABLE 2:** Procedural characteristics.

	Age < 75 years	Age ≥ 75 years	
	*N* = 499	*N* = 65	*p*
Ablation lesion set			
Pulmonary vein	498 (99)	65 (100)	0.72
Posterior wall	201 (40)	28 (43)	0.69
Superior vena cava	15 (3)	2 (3)	0.98
Number of PFA applications			
Total applications	58 (50–70)	56 (48–71)	0.90
Pulmonary vein applications	50 (48–56)	48 (46–52)	0.66
Posterior wall applications	22 (18–24)	20 (18–26)	0.65
Superior vena cava applications	4 (4–5)	6 (6–6)	0.06
Procedural time, minutes	65.8 ± 21.5	61.6 ± 22.6	0.15
Left atrial catheter dwell time, minutes	42.6 ± 15.8	40.9 ± 14.1	0.44
Application time, minutes	27.6 ± 10.8	26.7 ± 9.4	0.55
Fluoroscopy time, minutes	19.2 ± 9.3	18.0 ± 9.1	0.34
Radiation dose, Air Kerma mGy	153.8 (94.7–254.3)	123.4 (80.6–202.7)	0.53
Radiation dose, Dose‐area product μGym^2^	1676 (1018–2758)	1317 (932–1970)	0.35

Abbreviation: PFA, pulsed field ablation.

### 3.3 Procedural Complications

Older patients were more likely to have planned overnight admission (33/65; 51%) compared with younger patients (170/499; 34%; *p* = 0.008). There was no difference in the overall rate of major procedural complications between the older and younger cohorts (1.5% and 3.1%, respectively; *p* = 0.40). There were no cases of injury to non‐cardiac structures such as atrio‐esophageal fistula or phrenic nerve injury, and no cases of symptomatic pulmonary vein stenosis. However, patients in the older group had an increased risk of major vascular complications compared with younger patients (3.1% and 0.2%, respectively; *p* = 0.003). A 70 year old female had haematoma involving the right rectus sheath post‐ablation requiring hospital admission which was conservatively managed. A 79 year old female had percutaneous suture device closure (ProGlide) to close both the 16.8Fr (Faradrive) sheath and 6Fr sheath; however, she was noted to have inadvertent femoral arterial injury post‐ablation requiring surgical exploration and repair. A 75 year old male required an unplanned overnight hospital admission after developing hypotension post‐ablation with concern for groin haematoma which was conservatively managed and resolved without further intervention (Table 3).

**TABLE 3:** Procedural complications.

	Age < 75 years	Age ≥ 75 years	
	*N* = 499	*N* = 65	*p*
Major procedural complications (%)	8 (1.6)	2 (3.1)	0.40
Death	2 (0.4)	0	0.61
Atrio‐esophageal fistula	0	0	N/A
Symptomatic pulmonary vein stenosis	0	0	N/A
Cardiac tamponade	2 (0.4)	0	0.61
Stroke	4 (0.8)	0	0.47
Coronary artery spasm	0	0	N/A
Persistent phrenic nerve injury	0	0	N/A
Major vascular complication	1 (0.2)	2 (3.1)	**0.003**
Dialysis requirement	1 (0.2)	0	0.72
Minor procedural complications (%)	60 (12)	10 (15)	0.44
Systemic air embolism	0	0	N/A
Transient ischaemic attack	3 (0.6)	0	0.39
Transient phrenic nerve injury	0	0	N/A
Minor vascular complication	40 (8.0)	5 (7.7)	0.92
Pericarditis	13 (2.6)	1 (1.5)	0.60
Pulmonary oedema	2 (0.4)	2 (3.1)	**0.02**
Haemolysis	1 (0.2)	0	0.72
Transient renal impairment	4/38 (11)	4/8 (50)	**0.007**

*Note:* Bold values indicate statistically significant values.

There was no difference in overall rate of minor procedural complications (*p* = 0.44). However, patients in the older group had a higher incidence of pulmonary oedema (3.1% versus 0.4%; *p* = 0.02). In the two older patients (aged 75 years and 76 years), they both had preserved LV systolic function although dilated atria (both 50 mL/m^2^) and had received at least 2 L of intravenous fluid throughout the procedural period. In 46 patients who had their renal function assessed day one post‐procedure based on clinical concern, transient renal impairment was more common in older patients (4/8 [50%] versus 4/38 [11%]; *p* = 0.007).

### 3.4 Clinical Outcomes

Arrhythmia follow‐up data were available in 308 patients aged < 75 years (62%) and 41 patients (63%) aged ≥ 75 years (*p* = 0.83 between groups). In 342 patients who had documented follow‐up for assessment of arrhythmia recurrence, there was no significant difference between the older and younger cohorts (26.8% and 30.2%, respectively; *p* = 0.66) at median follow‐up of 7.0 months (IQR 1.9–14.7) and 6.3 months (IQR 2.7–13.4), respectively (Figure 1). Twenty‐four (4.2%) patients had an implantable cardiac device or Holter monitor during follow‐up. There was no difference detected in arrhythmia recurrence between patients who had an implantable cardiac device or Holter monitor (30.2%) compared with those who did not undergo any continuous rhythm monitoring (21.7%) during follow‐up (*p* = 0.09). Statistical evaluation of rates of arrhythmia recurrence according to the availability of electroanatomic mapping between groups could not be performed due to insufficient cases. However, the rate of subsequent cardioversions was higher in the older cohort (11% versus 4.6%; *p* = 0.04). There was no difference in the rate of hospitalization related to arrhythmia recurrence between the older and younger cohorts (4.6% versus 2.4%, respectively; *p* = 0.30) or redo‐procedure (6.2% in both groups; *p* = 0.98).

## 4 Discussion

We conducted a retrospective analysis of consecutive patients undergoing PFA for AF comparing procedural safety and clinical outcomes between patients aged less than 75 years and those 75 years and over. We found

1. No difference in ablation lesion set performed and procedural time between groups.
2. Increased risk of major vascular complications (3.1% vs. 0.2%), pulmonary oedema (3.1% vs. 0.4%), and transient renal impairment in older patients
3. No significant difference in arrhythmia recurrence, hospitalizations, or redo procedures; however, an increased proportion of subsequent cardioversions in the older cohort (11% vs. 4.6%).

Pulsed field ablation has emerged as a form of catheter ablation for AF associated with reduced procedural time, left atrial dwell time and similar clinical outcomes compared with traditional thermal ablation [11]. Given the improved procedural workflow and less concern for injury to surrounding non‐cardiac structures, it is likely that there will be a reduced threshold to offer an ablation to patients of various demographics [15]. Older patients have historically been excluded or under‐represented in clinical trials. In our study, the median age of patients in the older group was 77 years compared with 62 years in the younger group. The older cohort had expectedly higher CHA_2_DS_2_‐VASc (3.6 vs. 1.7), explained inherently by age but also higher incidence of hypertension and vascular disease, as well as lower eGFR. Thus older patients represent an inherently higher risk cohort. Nevertheless, similar proportions of patients in the younger and older cohort had persistent AF (37%–40%) with predominantly normal left ventricular systolic function and similar indexed left atrial volumes (44.1–45.4 mL/m^2^). From a procedural workflow perspective, there was no significant difference in the type of lesion set performed and the procedural and left atrial dwell time between groups. A study comparing outcomes in patients > 75 years between different catheter modalities (PFA vs. RFA vs. cryoballoon) similarly found that PFA was associated with shorter procedural times and more extra‐pulmonary vein lesion sets [16], which likely reflects proceduralist comfort with using PFA without causing significant extra‐cardiac collateral harm.

There was no significant difference in the rate of overall major procedural complications between the older and younger cohort in our study. In the EU‐PORIA registry concerning the pentaspline PFA catheter, there were comparable rates of overall complications (5.7% vs. 3.7%) in older (> 80 years) compared with younger patients, although they noted an increased incidence of stroke in older patients (2.3% vs. 0.3%) despite mostly minimally interrupted anticoagulation [17]. In our study, there was a statistically significant greater number of major vascular complications in the older cohort. This is consistent with data from several meta‐analyses evaluating thermal catheter ablation in older patients aged > 75–80 years that reported higher procedural complications in older patients compared with younger patients [18, 19]. It is not unexpected that vascular access complications are higher in the older patients in view of their relatively increased frailty and increased predisposition to vasculopathy. A randomized trial showed that femoral bleeding complications from PFA may be reduced with a percutaneous vascular closure device [20]. Nevertheless, it should be noted that there remained no overall difference in major procedural complications nor minor vascular complications [21]. However, this remains hypothesis‐generating data, and operators may be biased as older patients were more likely to have planned overnight admission compared with younger patients. Pulmonary oedema was statistically more common in the older patients and likely reflects the comorbid presence of heart failure with preserved ejection fraction phenotype and AF observed in elderly patients [22]. It is notable that both patients in the older cohort who developed pulmonary oedema had severely dilated left atria with preserved left ventricular systolic function. Additionally, our institutional practice has previously been generous with intravenous fluid administration to reduce the risk of intravascular haemolysis and acute renal impairment. We have since adopted a more judicious approach to intravenous fluid administration given the associated risks of haemolysis tend to be subclinical in our experience. Transient renal impairment was also detected more frequently in the older patients; however, this is subject to selection bias given that renal function was evaluated only where there was clinical concern in predominantly hospitalized patients.

There was no difference in arrhythmia recurrence at median follow‐up of 6.3–7.0 months in our study. Data from observational studies suggest an increased risk of arrhythmia recurrence compared with younger patients [18, 19]. While there was a greater proportion of older patients who underwent subsequent cardioversion, rates of overall hospitalizations were similar. Data comparing PFA with RFA and cryoballoon ablation in older patients showed comparable clinical outcomes with PFA [16]. Additionally, lesion durability at longer follow‐up with PFA remains uncertain, with pulmonary vein reconnection observed in 57% of patients undergoing repeat procedure for arrhythmia recurrence with this pentaspline catheter [23].

We acknowledge several limitations in this study. First, this is a retrospective analysis of consecutive patients undergoing PFA of which 11.5% were patients ≥ 75 years. There is likely selection bias, particularly in older patients, who may have been referred and deemed appropriate for AF ablation. Second, follow‐up data for arrhythmia recurrence was only available in 62% of patients predominantly due to private cardiologist follow‐up. Third, arrhythmia recurrence was assessed clinically and with 12‐lead electrocardiogram at clinic review. Systematic monitoring (e.g., Holter) was not routinely performed post‐procedure for otherwise asymptomatic patients. There is possibility of under‐reporting of arrhythmia recurrence. Fourth, arrhythmia follow‐up data are relatively short, although may appear artificially shorter as the first arrhythmia recurrence was taken as the last follow‐up. Fifth, specific complications such as transient renal impairment were not routinely assessed and would only be assessed where there was clinical concern. Thus the rate of transient renal impairment and other serum biomarkers of potential complications such as haemolysis may be under‐estimated. Lastly, these procedural and clinical outcomes relate to a specific PFA pentaspline catheter and how outcomes compare with other PFA catheter platforms are not yet known.

## 5 Conclusion

Pulsed field ablation for AF can be performed in older patients aged more than 75 years with acceptable procedural safety and clinical outcomes. Longer term follow‐up and clinical trials are needed to prospectively evaluate the safety and efficacy of this increasingly performed catheter ablation modality.

## Funding

F.J.H has received a postgraduate scholarship from the National Health and Medical Research Council and National Heart Foundation. No other funding was received.

## Disclosure

E.K. reports serving on the medical advisory boards for Medtronic, Boston‐Scientific, and Biotronik. Other authors do not have relevant disclosures.

## Ethics Statement

Ethics approval was granted by the institution's ethics review committee.

## Conflicts of Interest

E.K. reports serving on the medical advisory boards for Medtronic, Boston‐Scientific, and Biotronik. Other authors do not have relevant disclosures.

## Data Availability

The data that support the findings of this study are available from the corresponding author upon reasonable request.
